# Tropical Aquatic Skin and Soft Tissue Infections: A Series of Three Cases

**DOI:** 10.7759/cureus.13170

**Published:** 2021-02-06

**Authors:** Balram Rathish, Shazia M Mohammed, Krithi Ullal, Shafeeqa Hassan, Arun Wilson

**Affiliations:** 1 Infectious Diseases, Aster Medcity, Kochi, IND; 2 Internal Medicine, Aster Medcity, Kochi, IND; 3 Dermatology, Lourdes Hospital, Kochi, IND

**Keywords:** aquatic infections, vibrio vulnificus, shewanella algae, mycobacterium marinum

## Abstract

Bacterial infections following aquatic exposure occur frequently and most commonly present as skin and soft tissue infections (SSTI). Aquatic SSTI bacterial infections are usually caused by a limited number of organisms. Here we present three cases from the same geographical region, caused by three different organisms in patients who had exposure to an aquatic environment: Mycobacterium marinum, Shewanella algae, and Vibrio vulnificus. We wish to highlight that aquatic bacterial infections can cause varying degrees of morbidity and even mortality. Each of these three cases represents an aquatic, tropical SSTI with a delayed diagnosis, most likely as a result of the lack of widespread awareness about these organisms.

## Introduction

Bacterial infections following aquatic exposure occur frequently and most commonly present as skin and soft tissue infections (SSTI). Even though the causative agents are numerous, the initial presentation of the SSTI may be similar to wound infections caused by non-aquatic bacteria in the form of cellulitis, necrotising infections, impetigo, or erysipelas [1]. Aquatic SSTI bacterial infections are usually caused by a limited number of organisms including Aeromonas hydrophila, Chromobacterium violaceum, Edwardsiella tarda, Erysipelothrix rhusiopathiae, Mycobacterium fortuitum, Mycobacterium marinum, Shewanella species, Streptococcus iniae, and Vibrio vulnificus [1].

Infected or contaminated wounds, as well as all puncture wounds, should ideally be cultured or biopsied immediately. The initial empirical antibiotic approach should include coverage of potential aquatic bacteria and definitive antimicrobial therapy should be decided based on the precise pathogen identification and antimicrobial susceptibility testing [1]. Here we present three distinct infections from the same geographical area, caused by three different organisms in patients who had exposure to an aquatic environment.

## Case presentation

Case one: Mycobacterium marinum

**Figure 1:**
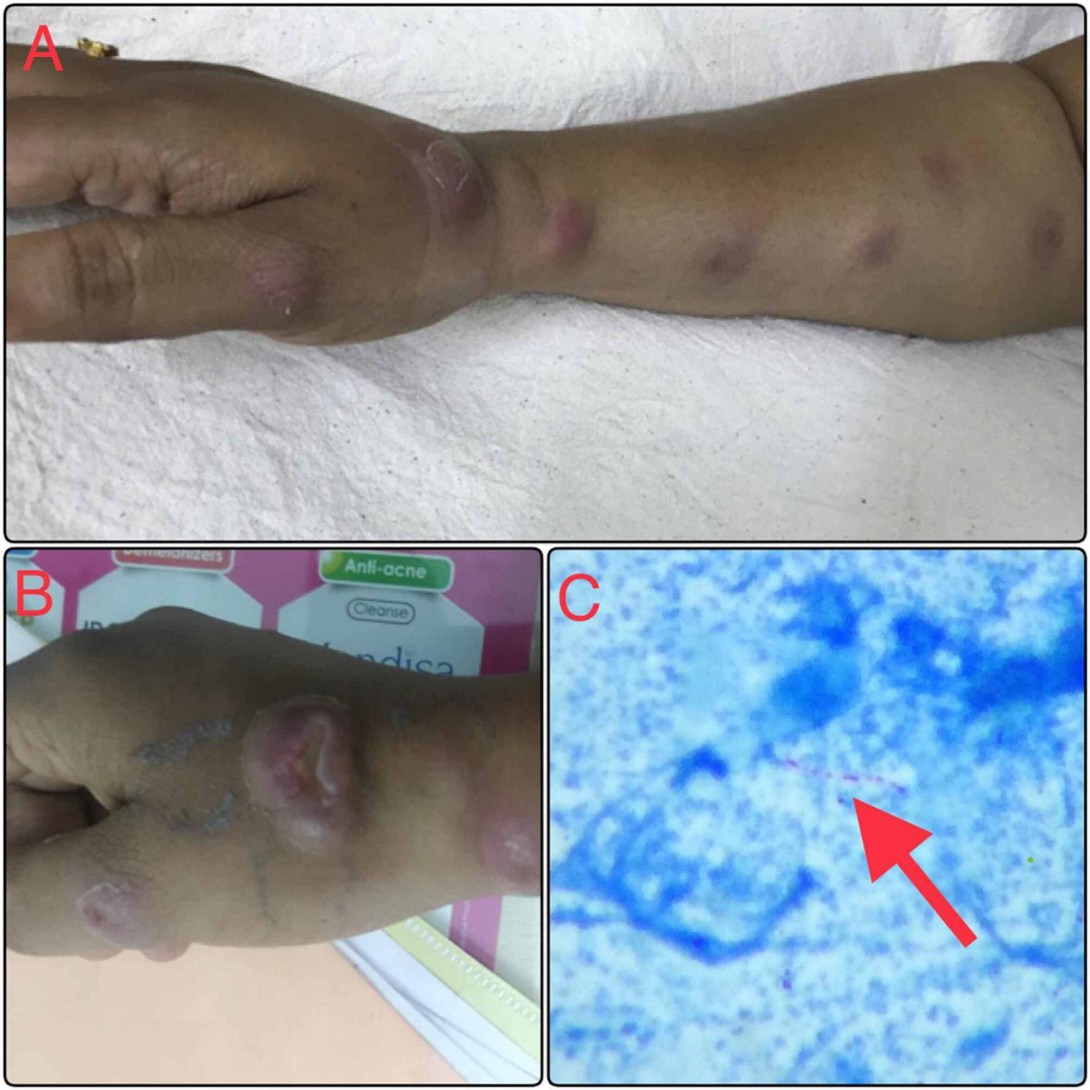
A. Sporotrichoid pattern of nodular rashes; B. Large nodule which had ulcerated; C. Modified Ziehl-Neelsen staining showing acid-fast bacilli (red arrow)

A 30-year-old lady with no co-morbidities presented with multiple rashes over her right forearm, two weeks after sustaining a cut injury to the right wrist while cleaning a fishbowl. The rash initially began on the wrist, followed by similar raised rashes developing along the length of her right forearm up to her elbow. She was treated with itraconazole from elsewhere for suspected Sporothrix infection, but one rash progressed to become a swelling and she was then referred to us. Examination showed multiple nodules in a sporotrichoid pattern with a large nodule that had formed an abscess (Figure 1). Blood investigations were unremarkable. Skin biopsy taken from the biggest nodular lesion showed only inflammatory changes. Gram staining, potassium hydroxide (KOH) smear, and bacterial culture were negative. Modified Ziehl-Neelsen staining revealed acid-fast bacilli (Figure 1). GeneXpert® (Cepheid, California, United States) for tuberculosis was negative, but Mycobacterium marinum polymerase chain reaction (PCR) was positive. She was started on rifampicin and clarithromycin following which she improved clinically and her lesions healed completely.

Case two: Shewanella algae

**Figure 2:**
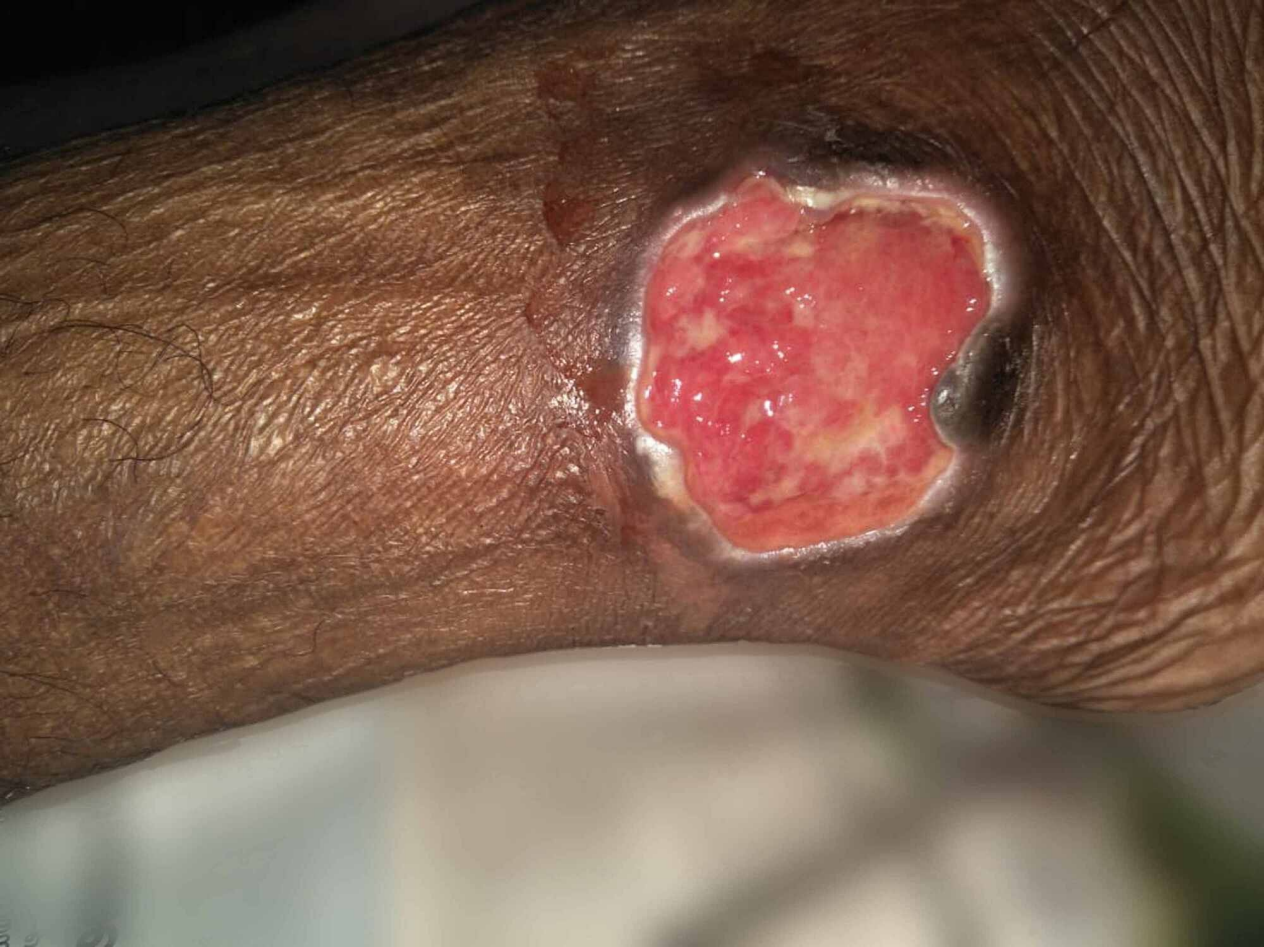
Large non-healing ulcer over the lateral malleolus

A 46-year-old lady with long-standing rheumatoid arthritis on methotrexate from a coastal area presented with a non-healing ulcer over the right lower leg for the past five months. She had repeated exposure to brackish water by virtue of walking through such areas where she lived. On examination, there was an ulcer over the right lateral malleolus (Figure 2). Workup showed raised inflammatory markers. She was treated with 300 mg twice daily oral clindamycin empirically. Bacterial culture of wedge biopsy tissue from the wound grew Shewanella algae which was identified using Vitek® 2 GN ID card (BioMérieux, Marcy-l'Étoile, France). It was found to be susceptible to aminoglycosides, quinolones, cephalosporins, and co-trimoxazole. Her antibiotic was changed to 750 mg once daily oral levofloxacin for 14 days following which she improved symptomatically. A wound debridement with local flap cover and skin grafting was planned after the complete resolution of the infection. 

Case three: Vibrio vulnificus

**Figure 3:**
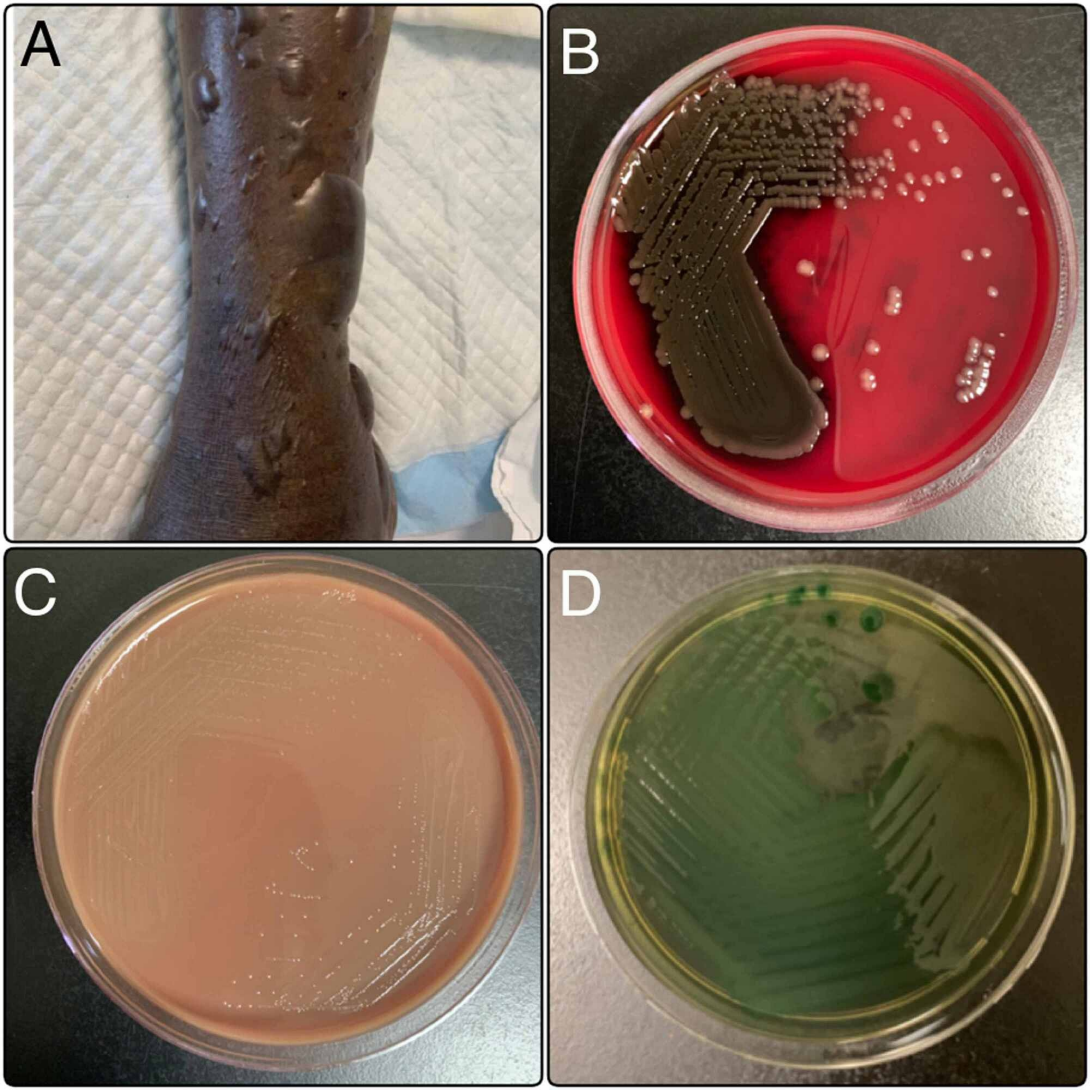
A. Multiple large blisters over the lower limb; B. Blood agar plate showing grayish white and glistening colonies suggestive of Vibrio; C. MacConkey agar plate showing non-lactose fermenting colonies suggestive of Vibrio; D. Thiosulfate citrate bile salts sucrose agar showing significant growth of green colonies consistent with Vibrio vulnificus

A 63-year-old man with poorly controlled diabetes and alcoholic liver disease was admitted with two days of fever and painful swelling over both lower limbs. The swelling was initially generalized, with the formation of small blisters at the time of admission. He was a fisherman and had waded through seawater repeatedly in the past week. Examination showed features of systemic inflammatory response syndrome and cellulitis of bilateral lower limbs. Workup showed high blood sugar, elevated inflammatory markers, deranged liver functions, and lactic acidosis. A chest X-ray was normal. He was started on intravenous piperacillin-tazobactam and oral clindamycin after obtaining blood for culture. Four hours later, the blood culture was indicative of the presence of gram-negative bacilli (GNB) (Figure 3). Piperacillin-tazobactam was changed to meropenem and doxycycline was added to cover for tropical infections. He developed hypotension and worsening acidosis requiring inotropic support and mechanical ventilation. The blisters on his legs enlarged and the erythema had extended proximally up to mid-thigh (Figure 3). He required urgent surgical debridement but developed a sudden cardiac arrest and succumbed. The GNB was later identified to be Vibrio vulnificus sensitive to ceftriaxone, levofloxacin, and minocycline. 

## Discussion

Infection of an open wound in a marine or aquatic environment, or exposure to salty water coupled with the lymphatic nature of spread pointed towards Mycobacterium marinum as a possible causative organism in our first case [1,2]. She was initially treated for a Sporothrix schenkii infection where the preceding history of an aquatic exposure was either missed or not deemed significant. Sporothrix schenckii remains the most common cause of nodular lymphangitis. Histopathology and culture are recommended for lesions that do not respond to treatment for sporotrichosis. Less commonly, sporotrichoid pattern may be commonly encountered in infections caused by Mycobacterium marinarum, Leishmania brasiliensis, or Nocardia species among others [3]. Mycobacterium marinum is a well-described pathogenic non-tuberculous mycobacterium (NTM) which usually causes SSTIs, and is commonly associated with fish and aquatic environments. Mycobacterium chelonae and Mycobacterium fortuitum are other rapidly growing NTMs that can present in a sporotrichoid pattern [3]. 

In our second case, the culprit organism was Shewanella algae. Shewanella are saprophytic, motile, GNB, widely distributed in nature. Shewanella infections are associated with aquatic and marine habitats [1,4]. Although SSTI and wound infection due to Shewanella species have been reported from different geographical areas, reports from the Indian sub-continent are rare. This could be due to the misidentification of all gram-negative and oxidase-positive organisms as Pseudomonas species. Hence it becomes important to look for organisms like Shewanella especially in the clinical background of an aquatic exposure [5].

Our third case was caused by Vibrio vulnificus. Vibrio vulnificus is an opportunistic human pathogen that can cause wound infections contaminated by warm seawater which can later lead to necrotising fasciitis. Most patients with Vibrio vulnificus infection develop sepsis and cellulitis with rapid development of ecchymoses and bullae. The mortality is reported to be higher than 50% for primary septicemia, with death occurring within 72 hours of hospitalization [6]. Vibrio vulnificus infections are reported from the Americas, South and Eastern Asia, and Australia [7]; they are rarely reported from India [8]. Our patient had a history of alcoholic chronic liver disease which placed him at a high risk for the development of a severe infection with Vibrio vulnificus. This, coupled with his occupation of being a fisherman, which required him to constantly wade in seawater, put him at an amplified risk for such a severe infection. Even with prompt initiation of intravenous antibiotics on admission and the addition of doxycycline within a few hours of admission, the patient had a rapid deterioration, further highlighting the sheer invasiveness and virulence of the organism. 

## Conclusions

In conclusion, we wish to highlight that aquatic bacterial infections can cause varying degrees of morbidity and mortality. The clinical presentation of such infections are also varied, ranging from an acute presentation of cellulitis and blister formation to chronic non-healing ulcers. Each of these three cases represented an aquatic, tropical SSTI with a delayed diagnosis, most likely as a result of the lack of widespread awareness about these organisms amongst the medical fraternity. The clinical history of exposure to an aquatic source becomes important in similar cases and could help lead to an early diagnosis and prompt initiation of treatment. These cases serve to highlight the prevalence of these infections in the Indian subcontinent. A high degree of clinical suspicion is warranted in the clinical and microbiological workup of such cases.
